# Gastric mucosal status in populations with a low prevalence of *Helicobacter pylori* in Indonesia

**DOI:** 10.1371/journal.pone.0176203

**Published:** 2017-05-02

**Authors:** Muhammad Miftahussurur, Iswan Abbas Nusi, Fardah Akil, Ari Fahrial Syam, I. Dewa Nyoman Wibawa, Yudith Annisa Ayu Rezkitha, Ummi Maimunah, Phawinee Subsomwong, Muhammad Luthfi Parewangi, I. Ketut Mariadi, Pangestu Adi, Tomohisa Uchida, Herry Purbayu, Titong Sugihartono, Langgeng Agung Waskito, Hanik Badriyah Hidayati, Maria Inge Lusida, Yoshio Yamaoka

**Affiliations:** 1 Department of Environmental and Preventive Medicine, Oita University Faculty of Medicine, Yufu, Japan; 2 Department of Medicine, Gastroenterology and Hepatology Section, Baylor College of Medicine, Houston, Texas, United States of America; 3 Division of Gastroentero-hepatology, Department of Internal Medicine, Faculty of Medicine-Dr. Soetomo Teaching Hospital, Universitas Airlangga, Surabaya, Indonesia; 4 Institute of Tropical Disease, Universitas Airlangga, Surabaya, Indonesia; 5 Center of Gastroentero-hepatology, Department of Internal Medicine, Faculty of Medicine, Hasanuddin University, Makassar, Indonesia; 6 Division of Gastroenterology, Department of Internal Medicine, Faculty of Medicine, University of Indonesia, Jakarta, Indonesia; 7 Division of Gastroentero-hepatology, Department of Internal Medicine, Faculty of Medicine University of Udayana, Denpasar, Indonesia; 8 Department of Molecular Pathology, Oita University Faculty of Medicine, Hasama-machi, Yufu-City, Oita, Japan; International Centre for Diarrhoeal Disease Research Bangladesh (icddr,b), BANGLADESH

## Abstract

In Indonesia, endoscopy services are limited and studies about gastric mucosal status by using pepsinogens (PGs) are rare. We measured PG levels, and calculated the best cutoff and predictive values for discriminating gastric mucosal status among ethnic groups in Indonesia. We collected gastric biopsy specimens and sera from 233 patients with dyspepsia living in three Indonesian islands. When ≥5.5 U/mL was used as the best cutoff value of *Helicobacter pylori* antibody titer, 8.6% (20 of 233) were positive for *H*. *pylori* infection. PG I and II levels were higher among smokers, and PG I was higher in alcohol drinkers than in their counterparts. PG II level was significantly higher, whereas PG I/II ratios were lower in *H*. *pylori*-positive than in *H*. *pylori*-negative patients. PG I/II ratios showed a significant inverse correlation with the inflammation and atrophy scores of the antrum. The best cutoff values of PG I/II were 4.05 and 3.55 for discriminating chronic and atrophic gastritis, respectively. PG I, PG II, and PG I/II ratios were significantly lower in subjects from Bangli than in those from Makassar and Surabaya, and concordant with the ABC group distribution; however, group D (*H*. *pylori* negative/PG positive) was the lowest in subjects from Bangli. In conclusion, validation of indirect methods is necessary before their application. We confirmed that serum PG level is a useful biomarker determining chronic gastritis, but a modest sensitivity for atrophic gastritis in Indonesia. The ABC method should be used with caution in areas with a low prevalence of *H*. *pylori*.

## Introduction

*Helicobacter pylori* has a unique capacity to persistently colonize the extremely acidic environment of the stomach and cause progressive gastric mucosal inflammation. Long-term infection induces a multistep histological cascade, from chronic non-atrophic gastritis that progresses to chronic atrophic gastritis, intestinal metaplasia, and adenocarcinoma [1]. Currently, chronic atrophic gastritis characterized by chronic inflammation with loss of gastric glandular cells is an established precursor lesion to gastric adenocarcinoma [2]. Although gastric mucosal biopsy is the reference method for determining the grade and topographical distribution of gastritis [3], this method is uncomfortable and expensive for patients. In contrast, serology is a cheaper, acceptable, and easily repeated method. Recent reports confirmed that serum pepsinogens (PGs) are a valuable biomarker of the gastric mucosal status, including inflammation, atrophic gastritis, and gastric cancer [4], even before the discovery of *H*. *pylori* [5].

Although the most part of PGs are secreted by the gastric cells and, in low levels, are permeated into serum [6]. PG I and PG II, the two main types of PGs are produced in different regions of the stomach [7]. Serum PG I, which is secreted purely on the fundus, decreases progressively, whereas PG II level decreases less markedly or remain stable owing to the additional production in non-gastric glands such as the duodenal bulb; therefore, PG I/II ratios serve as a gastric mucosal biomarker [7] and can be applied to gastric cancer screening [4, 8, 9]. The cutoff points of ≤70 μg/L and ≤3 for PG I level and PG I/II ratio, respectively, were used in Japan for identifying the risk of gastric cancer [10]. However, the discriminative baseline PG levels may not be applicable to other countries and therefore should be recalculated, because several factors including geographic area, race, age, sex, smoking and drinking habits, and *H*. *pylori* infection also contribute to the levels [11–13]. In addition, there is evidence that PG II level increases and the PG I/II ratio decreases with the grade of inflammation [14], providing an opportunity for the application PG levels in patients with chronic gastritis who also frequent in countries with a lower prevalence of gastric cancer than that in Japan [10].

As we explained in detail previously [15–19], Indonesia is a multiethnic nation in Southeast Asia with a relatively low risk of gastric cancer (age-standardized incidence rate [ASR]: 2.8 in 100,000; GLOBOCAN2012, http://globocan.iarc.fr/) with the overall prevalence of *H*. *pylori* infection in the five largest islands of Indonesia was 22.1% [17]. The predominant ethnic, Javanese, has a low prevalence of *H*. *pylori* infection (2.4%) [16, 17], however, several ethnic groups have a much higher risk of *H*. *pylori* infection [17]. According to Indonesia as the fourth most populous country worldwide with a high prevalence of *H*. *pylori* antibiotic resistance [19, 20], and the strains harbor more virulent *H*. *pylori* genotypes [18], consequently, *H*. *pylori-*associated diseases are an important problem in Indonesia. Moreover, although dyspepsia was sixth and fifth of the 10 most prevalent outpatient and inpatient diseases in Indonesia, respectively [21], hospitals that provide gastrointestinal endoscopy services in the country are very limited. Therefore, noninvasive methods such as measurement of PG values are the best choice for determining the gastric mucosal status in Indonesia, especially in remote areas.

To our knowledge, studies examining the gastric mucosal status in Indonesia by using PG levels are rare [22, 23]. Two previous studies determined the accuracy of PG levels in a single city that was predominantly inhabited by the Javanese people: Purwokerto [22] and Jakarta [23]. These studies also did not compare the PG data with histological grades (e.g., updated Sydney system) [24]. The study performed in Purwokerto reported that the specificity of PG I level to predict atrophic chronic gastritis was only 50%, with a sensitivity of 70% [22]. In contrast, the study in Jakarta suggested a low sensitivity (43%) of PG I/II as the biomarker of *H*. *pylori* chronic gastritis, with a specificity of 83% [23]. In the current study, we measured the PG levels of three ethnic groups from different islands. We also calculated the best cutoff and predictive values for discriminating chronic and atrophic gastritis based on PG levels among ethnic groups in Indonesia.

## Materials and methods

### Study population

We performed a prospective study between January and August 2015 in Surabaya, Java Island; Makassar, Sulawesi Island; and Bangli, Bali Island. We excluded subjects with a history of *H*. *pylori* eradication therapy and partial/total gastrectomy, nonfasted subjects, and those with contraindication for upper endoscopy. Experienced endoscopists acquired two gastric biopsy specimens during each endoscopy procedure: one from the lesser curvature of the antrum approximately 3 cm from the pyloric ring, and another from the greater curvature of the corpus, which were used for histological examination. Fasting serum was collected on the day of endoscopy and then stored at -20°C. Socio-demographic data including body mass index, smoking and drinking habits, and use of nonsteroidal anti-inflammatory drugs (NSAIDs) were collected during the interview. Written informed consent was obtained from all participants, and the study protocol was approved by the ethics committees of Dr. Soetomo Teaching Hospital (Surabaya, Indonesia), Dr. Wahidin Sudirohusodo Teaching Hospital (Makassar, Indonesia), and Oita University Faculty of Medicine (Yufu, Japan).

### Determination of *H*. *pylori* serology and PG levels

The separated sera were used for the measurement of the *H*. *pylori* antibody titers and PG levels. The anti-*H*. *pylori* IgG levels were quantified by using an ELISA kit (Eiken, Co. Ltd., Tokyo, Japan), and the PG I and II levels were measured by using PG ELISA (Eiken), according to the manufacturer’s instructions. Subjects with serum *H*. *pylori* antibody titers ≥10 U/mL were classified as *H*. *pylori* positive per the manufacturer’s instructions. Those with PG I level ≤70 ng/mL and PG I/II ratio ≤3.0 were classified as PG positive according to the Japanese guidelines [4]. According to the ABC method, we categorized the subjects into four groups: *H*. *pylori* negative/PG negative (group A), *H*. *pylori* positive/PG negative (group B), *H*. *pylori* positive/PG positive (group C), and *H*. *pylori* negative/PG positive (group D) [4].

### Histology and immunohistochemistry

All biopsy materials for histological testing were fixed in 10% buffered formalin and embedded in paraffin. Serial sections were stained with hematoxylin and eosin and May–Giemsa stain. The degree of inflammation, atrophy, and bacterial density were classified into four grades according to the updated Sydney system: 0, normal; 1, mild; 2, moderate; and 3, marked [24]. Samples with bacterial loads ≥grade 1 were considered positive for *H*. *pylori*.

To increase the accuracy for detecting *H*. *pylori*, we performed immunohistochemical confirmation, as previously described [25]. Briefly, after antigen retrieval and inactivation of endogenous peroxidase activity, tissue sections were incubated with anti-α-*H*. *pylori* antibody (DAKO, Glostrup, Denmark) overnight at 4°C. After washing, the sections were incubated with biotinylated goat anti-rabbit IgG (Nichirei Co., Tokyo, Japan), followed by incubation with an avidin-conjugated horseradish peroxidase solution (Vectastain Elite ABC Kit; Vector Laboratories Inc., Burlingame, CA, USA). Peroxidase activity was detected by using an H_2_O_2_/diaminobenzidine substrate solution. To minimize potential bias, the same experienced pathologist (TU) who also performed experiments for Myanmar, Vietnam, Bhutan, Dominican Republic, and Indonesia [16, 26–30] evaluated all the specimens in this study.

### Data analyses

Discrete variables were tested by using the chi-square test; continuous variables were tested with the Mann–Whitney *U* and Kruskal–Wallis test. The Spearman rank coefficients (r) were determined to evaluate the association between PG levels and gastric mucosal inflammation and atrophy. A multivariate logistic regression model was used to calculate the ORs of the clinical outcomes by including age, sex, NSAID use, alcohol drinking, smoking, *H*. *pylori* infection, and gastritis type. All determinants with P < 0.10 were entered together in the full model of logistic regression, and the model was reduced by excluding variables with P > 0.10. The OR and 95% confidence interval (CI) were used to estimate the risk. P < 0.05 was accepted as statistically significant. Receiver-operating characteristic (ROC) curves were used to calculate the best cutoff, including the area under curve (AUC) and predictive values for discriminating chronic and atrophic gastritis. The SPSS statistical software package version 18.0 (SPSS Inc., Chicago, IL, USA) was used for all statistical analyses.

## Results

### *H*. *pylori* infection rate based on serology

We recruited 233 patients with dyspepsia comprising 110 women and 123 men with a mean age of 46.1 ± 12.3 years (range, 14–82 years), including 99 patients from Surabaya, 75 from Makassar, and 59 from Bangli (S1 Table). There were 19 patients with peptic ulcers. On the basis of histology confirmed by immunohistochemistry, 15 patients (6.4%) were positive for *H*. *pylori* infection.

**Fig 1:**
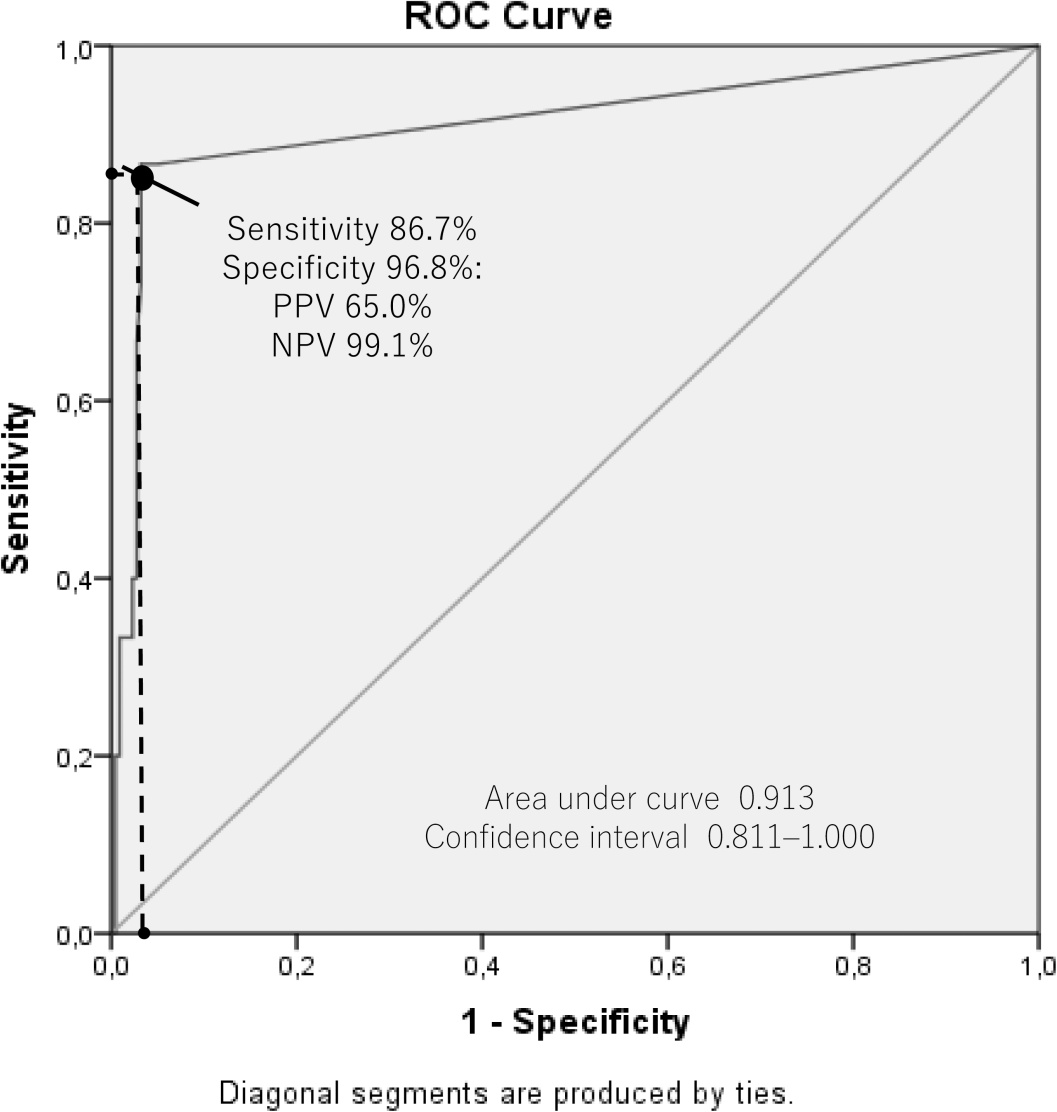
Receiver-operating characteristic (ROC) curve for determining the optimal cutoff of *Helicobacter pylori* antibody titers. The sensitivity delineated in X-axis (86.7%) and the Y-axis characterized value of 1-specificity (96.8%). PPV was a positive predictive value and NPV was a negative predictive value.

By using the cutoff value from the manufacturer’s instructions (positive if ≥10 U/mL), we found the sensitivity and specificity of the ELISA kit for *H*. *pylori* infection to be 66.7% and 97.2%, respectively, compared with histology confirmed by immunohistochemistry as a gold standard. As the low sensitivity was due to a large number of false negatives (5 of 15, 33.3%), we determined the best cutoff values of *H*. *pylori* antibody titers in ELISA kit with an ROC curve. We found that the cutoff of ≥5.5 U/mL was the best value to determine *H*. *pylori* positivity (sensitivity, specificity, positive predictive value [PPV], and negative predictive value [NPV]: 86.7%, 96.8%, 65.0%, and 99.1%, respectively; Fig 1), with an AUC of 0.913 (95% CI, 0.811–1.000). When we used the new cutoff value of ≥5.5 U/mL, 8.6% (20 of 233) were positive for *H*. *pylori* infection (S1 Table). The *H*. *pylori* infection rate by age group was 5.6% (1 of 18), 5.2% (3 of 58), 10.3% (6 of 58), 7.5% (5 of 67), and 15.6% (5 of 32) for patients aged ≤29, 30–39, 40–49, 50–59, and ≥60 years, respectively. Patients with peptic ulcer had a higher prevalence of *H*. *pylori* infection than those without ulcer (5 of 19, 26.3% vs. 15 of 214, 7.0%; P = 0.004). According to location, Bangli (13.6%, 8 of 59) and Makassar (12.0%, 9 of 75) had a higher prevalence of *H*. *pylori* infection than Surabaya (3.0%, 3 of 99) (P = 0.01 and 0.02, respectively).

### Socio-demographic data and PG levels

Women had lower PG I, PG II, and PG I/II ratio than men (P < 0.001, P < 0.001, P = 0.008, respectively). PG I and PG II were also increased with age (r = 0.34 and r = 0.32, P < 0.001, respectively), but not PG I/II ratio. There was no difference in the PG I and II levels and PG I/II ratios in patients with gastritis and those with peptic ulcer (P = 0.10, P = 0.08, P = 0.85, respectively). When we considered patients with any inflammation and atrophy in the antrum or corpus on histological examination as a symptomatic patient, PG II levels was higher and PG I/II ratio was lower among symptomatic than asymptomatic patients (15.4 ± 11.3 vs. 12.4 ± 9.6, P = 0.05 and 6.0 ± 1.8 vs. 6.6 ± 2.0, P = 0.017, respectively). There was no significant association between the PG levels and NSAID use and body mass index (P > 0.05). Smoking was significantly higher in subjects from Makassar and Bangli than in those from Surabaya (28.0% [21 of 75], 22.0% [13 of 59] vs. 10.1% [10 of 99], P = 0.002 and P = 0.04). PG I and II levels were higher in smokers than in nonsmokers (114.9 ± 87.2 vs. 79.1 ± 67.6, P = 0.001 and 18.9 ± 13.3 vs. 12.6 ± 9.4, P < 0.0001, respectively). There was no significant difference in drinking habits in all populations (P > 0.05). PG I level was also higher in drinking than in nondrinking subjects (111.8 ± 91.8 vs. 84.0 ± 70.9, P = 0.043).

### *H*. *pylori* infection status and PG levels

**Fig 2:**
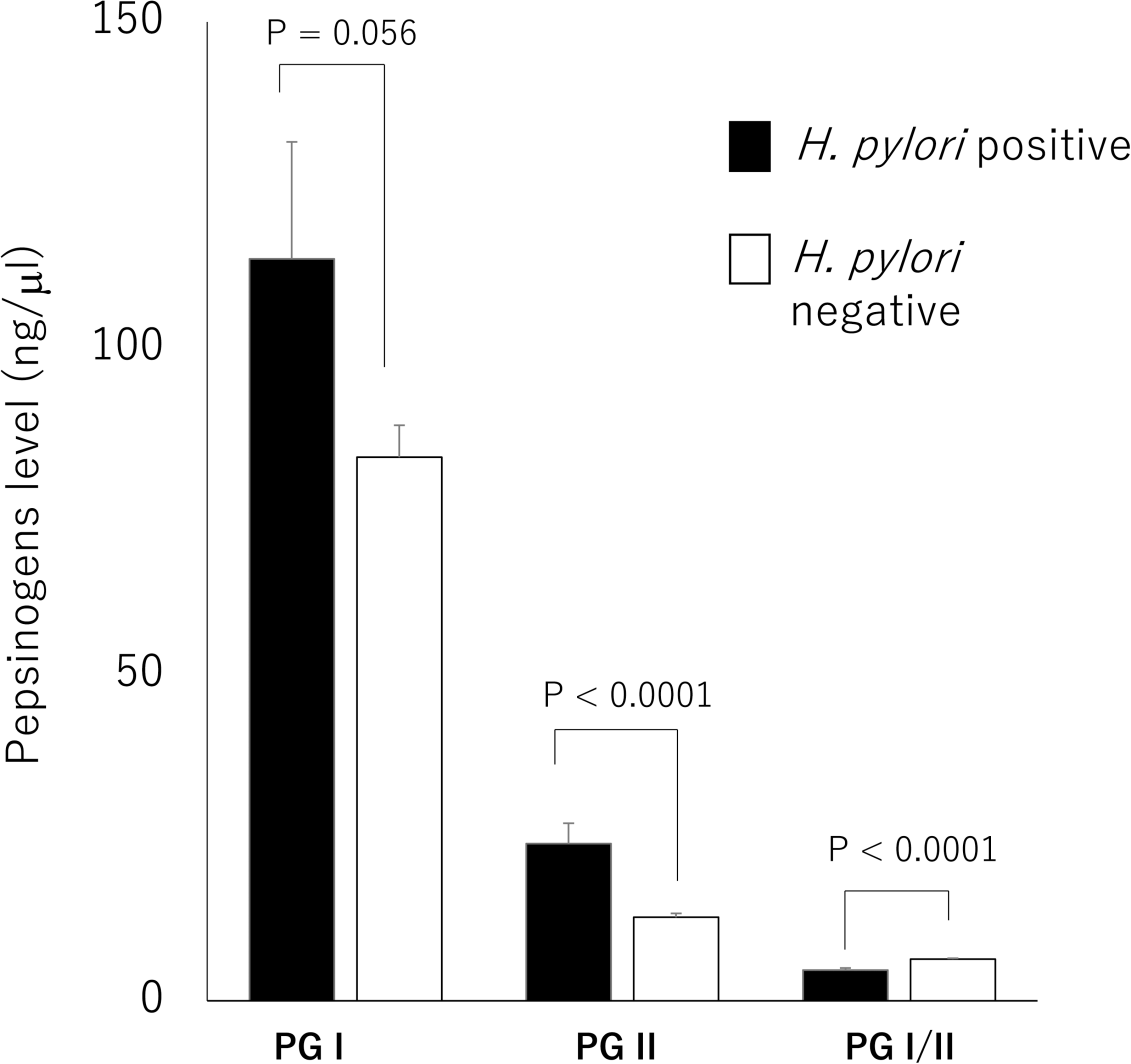
Pepsinogens levels among *H*. *pylori* positive and negative patients. PG II level was significantly higher and PG I/II ratios were significantly lower in *H*. *pylori*-positive than in *H*. *pylori*-negative patients.

PG I level tended to be higher in *H*. *pylori*-positive than in *H*. *pylori*-negative patients (P = 0.056). Additionally, PG II level was significantly higher in *H*. *pylori*-positive than in *H*. *pylori*-negative patients (P < 0.0001, Table 1), whereas the PG I/II ratios were significantly lower in *H*. *pylori*-positive than in *H*. *pylori*-negative patients (P < 0.0001, Fig 2). When we considered patients with any inflammation and atrophy in the antrum or corpus on histological examination as positive for chronic and atrophic gastritis, respectively, the prevalence of chronic and atrophic gastritis was higher in *H*. *pylori*-positive than in *H*. *pylori*-negative patients (both P < 0.0001). Only *H*. *pylori* infection was an independent risk factor for the development of chronic gastritis in the corpus but not in the antrum (OR, 13.12; 95% CI, 2.3–73.8) after adjustment for age, sex, NSAID use, alcohol drinking, and smoking. After adjustment, *H*. *pylori* infection also was an independent risk factor for the development of atrophy in the antrum (OR, 9.87; 95% CI, 1.8–54.4).

**Table 1:** Demographics and *Helicobacter pylori* antibody status (mean ± SD).

Parameter	*H*. *pylori* positive	*H*. *pylori* negative	P-value
Male/female	10/10	100/113	
Age	49.75 ± 12.1	45.80 ± 12.3	0.14
Body mass index (kg/m^2^)	21.82 ± 2.7	23.02 ± 3.8	0.18
Pepsinogen I (ng/mL)	113.73 ± 80.1	83.28 ± 71.8	0.056
Pepsinogen II (ng/mL)	24.09 ± 13.9	12.83 ± 9.6	<0.0001
Pepsinogen I/II ratio	4.70 ± 1.4	6.43 ± 1.9	<0.0001
Chronic gastritis (%)	20/20 (100.0)	69/213 (32.4)	<0.0001
Atrophic gastritis (%)	15/20 (75.0)	45/213 (21.2)	<0.0001

### PG levels and chronic gastritis

Inflammation in the antrum had a correlation with atrophy, similar to that in the corpus (P <0.001, r = 0.382 and P <0.001, r = 0.384, respectively). We examined the correlation between the severity of chronic gastritis and PG levels (Table 2). Overall, in patients with chronic gastritis, PG I level was almost stable in conjunction with the increase of PG II level and decrease of PG I/II ratio (P < 0.05). Although PG I level did not correlate with the inflammation scores (P = 0.55 in the antrum and P = 0.31 in the corpus), PG II level showed a weak correlation in the antrum and corpus (both P = 0.01, r = 0.16) and the association was disappeared in the multivariate analysis after adjusted with age, sex, smoking, drinking habits and positivity of *H*. *pylori* (P = 0.21). Additionally, PG I/II ratios showed a significant inverse correlation with the inflammation scores in the antrum and corpus (P < 0.0001, r = -0.24 and P = 0.001, r = -0.22, respectively). Moreover, when we analyzed only the *H*. *pylori*-positive patients, the PG I/II ratios showed a significant inverse correlation with the inflammation scores in the antrum (P = 0.02, r = -0.61) but not in the corpus (P = 0.52). In the multivariate analysis, the inverse association of PG I/II ratios and antral inflammation was still significant although adjusted with age, sex, smoking consumption and drinking habits (OR, 0.34; 95% CI, 0.21–0.56).

**Table 2:** Levels of pepsinogen (PG) I, PG II, and PG I/II in chronic and atrophic gastritis (mean ± SD).

	Grade	n	PG I	PG II	PG I/II
Chronic gastritis					
Antrum	0	147	85.3 ± 77.9	12.7 ± 9.7	6.5 ± 1.9
	1	68	89.3 ± 68.3	14.9 ± 11.4*	6.2 ± 2.0*
	2	13	89.7 ± 44.9	21.4 ± 13.2*	4.6 ± 1.4*
	3	5	46.7 ± 6.4	12.1 ± 2.8	4.1 ± 1.4
Corpus	0	187	84.5 ± 74.5	12.8 ± 9.7	6.4 ± 1.8
	1	42	90.2 ± 66.0	17.2 ± 12.7*	5.7 ± 2.4*
	2	4	106.5 ± 73.1	22.5 ± 13.5*	4.7 ± 2.0*
	3	0	NA	NA	NA
Atrophic gastritis					
Antrum	0	174	83.5 ± 72.0	12.8 ± 9.7	6.5 ± 2.0
	1	53	97.4 ± 78.5	17.2 ± 12.8*	5.7 ± 1.5*
	2	5	55.4 ± 10.8	13.6 ± 4.6	4.5 ± 1.8
	3	1	18	13.8	3.1
Corpus	0	224	84.6 ± 72.4	13.4 ± 10.2	6.3 ± 2.0
	1	8	112.9 ± 84.4	22.1 ± 13.8*	5.3 ± 1.9
	2	1	168.0	32.3*	5.2
	3	0	NA	NA	NA

* P < 0.05 vs. grade 0.

Next, we determined the best cutoff values for discriminating chronic gastritis. By using the ROC curve, PG II level was not found to be a good discriminatory marker for antral chronic gastritis (data not shown). For stages ≥II in the inflammation score, considered as positive for chronic gastritis, the best cutoff value of PG I/II was 5.65 (sensitivity, specificity, PPV, NPV, and accuracy: 83.3%, 63.7%, 16.1%, 97.9%, and 65.2%, respectively). The AUC was 0.832 (95% CI, 0.721–0.942). For stages ≥III in the inflammation score, the best cutoff value of PG I/II was 4.05 (sensitivity, specificity, PPV, NPV, and accuracy: 80.0%, 94.3%, 23.5%, and 99.5%, respectively). The overall accuracy was 94.0% with an AUC of 0.857 (95% CI, 0.664–1.000). The PG I best cutoff value was 47.5 (the AUC were 0.674 [95% CI, 0.585–0.762]) with sensitivity, specificity, PPV, NPV, and accuracy were 80.0%, 64.9%, 4.8%, 99.3%, 65.2%, respectively for stages ≥III in the inflammation score. When we used a combination PG I and PG I/II for stages ≥III in the antral chronic gastritis, the outcomes practically the same as when utilizing just PG I/II ratio although had a higher PPV than only PG I/II ratio marker (sensitivity, specificity, PPV, NPV, and accuracy were 80.0%, 97.3%, 40.0%, 99.6%, and 97.0%, respectively.

### PG levels and atrophic gastritis

According to the updated Sydney system, we determined that 74.3% of patients (173 of 233) had no mucosal atrophy in both the antrum and corpus, and these patients were categorized as the nonatrophic gastritis group. In addition, only 21.9% (51 of 233), 0.4% (1 of 233), and 3.4% (8 of 233) of patients had antral atrophy, corpus atrophy, and multifocal atrophy, respectively. There were differences in the PG II level and PG I/II ratio according to the pattern of atrophic gastritis (P = 0.009 and P = 0.023, respectively; Table 3). Compared with nonatrophic gastritis, antral atrophy had significantly lower PG/II ratio (P = 0.01). Moreover, PG I tended to have higher levels and PG I/II ratio tended to have lower levels in subjects with nonatrophic gastritis than in those with multifocal atrophic gastritis (P = 0.053 and P = 0.057, respectively). PG II had a significantly lower level in subjects with nonatrophic gastritis than in those with multifocal atrophic gastritis (P = 0.002).

**Table 3:** Pepsinogen level according to atrophic gastritis pattern.

Pepsinogens	No Atrophy	Antral atrophy	Corpus atrophy	Multifocal atrophy	P-value
N	173	51	1	8	
Pepsinogen I (ng/mL)	83.72 ± 72.1	87.42 ± 73.9	133.0	128.41 ± 81.3	0.24
Pepsinogen II (ng/mL)	12.79 ± 9.7	15.53 ± 11.7	17.9	25.30 ± 12.6*,**	0.009
Pepsinogen I/II ratio	6.51 ± 2.0	5.67 ± 1.5*	7.4	5.01 ± 1.9	0.023

Multifocal atrophy means the subjects had atrophy in both the antrum and corpus.

* P < 0.05 vs. no atrophy.

** P < 0.05 vs. antral atrophy.

Although the PG I level did not correlate with the atrophy scores (P = 0.49 in the antrum and P = 0.11 in the corpus), the PG II level and PG I/II ratios showed a significant correlation in the antrum (P = 0.02, r = 0.16 and P = 0.002, r = -0.20, respectively; Table 2) and only PG II showed a significant correlation in the corpus (P = 0.02, r = 0.16). However, there was no correlation between the PG levels and atrophy scores among *H*. *pylori*-positive patients. This may be due to the low number of *H*. *pylori-*positive patients with atrophic gastritis. When we analyzed only *H*. *pylori*-negative patients, the PG I and PG II levels showed a weak correlation with the atrophy scores in the corpus (P = 0.04, r = 0.14 and P = 0.03, r = 0.15, respectively).

When we used the Japanese standards to define the PG-positive status (cutoff values: ≤70 ng/mL and ≤3.0 for PG I level and PG I/II ratio, respectively), we found that the overall prevalence of PG-positive subjects was very low (1.3%, 3 of 233). Furthermore, when we used the cutoff value only for PG I/II ratios ≤3.0, the sensitivity, specificity, PPV, NPV, and accuracy were 5.1%, 99.4%, 75.0%, 75.6%, and 75.5%, respectively, for antral atrophic scores ≥1. In cases with an antral atrophic score ≥2, the values were 16.7%, 98.7%, 25.0%, 97.8%, and 96.6%, respectively.

Owing to the low sensitivity of the Japanese standard, we determined the best cutoff value of PG I/II ratio with an ROC curve. For stages ≥I in the antral atrophic score, considered as atrophy positive, the best cutoff value of PG I/II ratio was 6.05 (sensitivity, specificity, PPV, NPV, and accuracy: 61.0%, 55.2%, 31.6%, 80.7%, and 56.7%, respectively). The AUC was 0.629 (95% CI, 0.544–0.713). For stages ≥II in the antral atrophic score, considered as atrophy positive, the best cutoff value of PG I/II was 3.55 (sensitivity, specificity, PPV, NPV, and accuracy: 66.7%, 96.5%, 33.3%, 99.1%, and 95.7%, respectively), and the AUC was 0.793 (95% CI, 0.580–1.000).

The PG I and PG II best cutoff value for stages ≥II in the antral atrophic score were 65.0 (the AUC were 0.583 [95% CI, 0.482–0.684] with sensitivity, specificity, PPV, NPV, and accuracy was 83.3%, 47.1%, 96.0%, 99.1%, 48.1%, respectively. while PG II level was not observed to be a sufficient marker for antral chronic gastritis by using the ROC curve (data not shown). When we used a combination PG I and PG I/II ratio for stages ≥II in the antral atrophic score, the results almost the same as when using only PG I/II ratio (sensitivity, specificity, PPV, NPV, and accuracy were 66.7%, 97.8%, 44.4%, 99.1%, and 96.9%, respectively).

### PG levels and the ABC method among ethnic groups

The comparison of PG levels among the ethnic groups is shown in Table 4. Among the *H*. *pylori*-negative subjects, PG I and II levels were significantly lower in subjects from Bangli than in those from Makassar (both P <0.001). Furthermore, the PG I/II ratios were significantly lower in subjects from Bangli than in those from Makassar (P = 0.03). Subjects from Bangli also had significantly lower PG I/II ratios than those from Surabaya (P = 0.004). Although the PG I and II levels in subjects from Surabaya were significantly lower than in those from Makassar, the difference in PG I/II ratios was not significant (Table 4). The similar results also showed among the *H*. *pylori*-positive subjects, PG I and II levels were significantly lower in subjects from Bangli than in those from Makassar (56.3 ± 18.8 vs. 165.4 ± 87.6, P = 0.007 and 14.5 ± 6.5 vs. 31.6 ± 14.3, P = 0.012, respectively). However, there was no difference for the PG I/II ratios and PGs levels between Surabaya and Makassar subjects.

**Table 4:** Characteristics of pepsinogens among the ethnic groups.

Pepsinogens	Surabaya	Makassar	Bangli	P* value	P** value	P*** value
N	96	66	51			
PG I (ng/mL)	79.5 ± 58.1	113.2 ± 96.1	61.3 ± 54.2	0.01	0.07	<0.001
PG II (ng/mL)	12.0 ± 8.1	17.3 ± 13.0	10.6 ± 9.0	0.004	0.36	<0.001
PG I/II ratio	6.6 ± 1.8	6.5 ± 2.4	5.9 ± 1.3	0.56	0.004	0.03

PG, pepsinogen.

* Surabaya vs. Makassar.

** Bangli vs. Surabaya.

*** Bangli vs. Makassar.

By using ≥5.5 U/mL and ≤3.55 as the best cutoff values of *H*. *pylori* antibody titers and PG I/II ratio, respectively, we determined the distribution of the four groups in each age category, according to the ABC method. Overall, group A (207 of 233, 88.8%) was the most predominant, followed by groups B (14 of 233, 6.0%), C (6 of 233, 2.6%), and D (6 of 233, 2.6%). A comparison of the ABC method among the ethnic groups is shown in Table 5. Consistent with the results obtained for PG I/II ratios, subjects from Bangli had the highest proportion of group C and lowest proportion of group A compared with those from Makassar and Surabaya. Interestingly, the percentage of group D in subjects from Bangli was the lowest among all groups.

**Table 5:** ABC method among ethnic groups.

ABC groups	Surabaya (%)	Makassar (%)	Bangli (%)
Group A	94/99 (94.9)	63/75 (84.0)	50/59 (84.7)
Group B	3/99 (3.0)	7/75 (9.3)	4/59 (6.8)
Group C	0/99 (0.0)	2/75 (2.7)	4/59 (6.8)
Group D	2/99 (0.2)	3/75 (4.0)	1/59 (1.7)

## Discussion

Although the ELISA kit for serology, which was developed by using Japanese *H*. *pylori* strains, it reached a high sensitivity and specificity when used in Japan and Nepal (95.2–100% and 76.2–80.0% [31] and 89.1% and 83.5% [32], respectively), we confirmed the low accuracy of the ELISA kit when used in the Indonesian population. Therefore, we calculated the best cutoff values of IgG ELISA. The same kits also showed a low sensitivity in Myanmar [33] and a low specificity when used in the Bhutan population [34]. *H*. *pylori* antibody titers vary greatly depending on the test kit used [35], and in general showed a lower performance in general population, but an outstanding result on the selected samples [36]. The discrepancy of *H*. *pylori* positivity number using ELISA and histology-immunohistochemistry associated with the advantages and disadvantages of several tests, thus the selection of the test should be performed based on these considerations [37, 38]. Importantly, the low PPV of ELISA indicated that although the kits could become the first diagnostic test, it should be confirmed with other test in positive case, especially with a high PPV value test. The Maastricht III and IV consensus suggested that “some serological tests with good sensitivity and specificity can be used to perform the initial diagnosis of infection with *H*. *pylori*” [39], and only validated commercial tests should be used [38, 40]. Thus, an appraisal of validation has to be performed on every serology test to the particular study population, and the antigen has to be wisely selected [41]. It might be important to develop ELISA kits using native *H*. *pylori* strains to the study population.

Similar to our results, previous studies [42, 43] reported that PG levels have a positive correlation with aging, particularly in men, which may be related to the increase of renal function loss and influence of hormones [44, 45]. In concordance with our data, smoking was reported to stimulate PG secretion [13]. However, long-term stimulation may exhaust the chief cells and promote atrophy of the gastric mucosa, reflected as lower PG I/II ratios in smokers [46]. In our study, drinking habits increased the PG I and II levels, in contrast to previous results [13]. Our results also confirmed the involvement of *H*. *pylori* as a key factor in the development of chronic and atrophic gastritis even after adjustment for other factors. *H*. *pylori* infection induces mucosal inflammation and the lipopolysaccharide of bacteria directly stimulates PG secretion [47], which significantly decreases after eradication concomitant with the improvement in inflammation scores [48]. The stimulation is mediated by calcium, which increases histamine and dibutyryl-cyclic adenosine monophosphate [49].

Several studies suggested PG II as a good marker of gastric inflammation [45, 48, 50, 51]. Our study also showed the association between PG II and inflammation scores; however, it showed a weak correlation and was not a good discriminating marker for chronic gastritis. On the other hand, PG I/II ratios showed a high accuracy in predicting chronic gastritis, in line with previous results [14]. As Indonesia is a large country consisting of thousands of islands with a lack of endoscopy facilities, the validated PG I/II ratios offer the benefit of discriminating Indonesian patients with chronic gastritis. In addition, the PG I/II ratios also could discriminate antral atrophic gastritis, although with a lower sensitivity than that for chronic gastritis and the combination with PG I showed almost similar results. Although, overall, the PG I/II ratios had a high accuracy for predicting chronic and atrophic gastritis, they had low PPVs (23.5% and 33.3%, respectively) and high NPVs. It is suggested that PG I/II ratios have the advantages of excluding patients with chronic or atrophic gastritis (i.e., a negative result is usually a true negative result). However, the modest sensitivity result reduced the role of PG I/II ratio as valuable marker for atrophic gastritis screening. In contrast, a positive PG I/II result should be confirmed by using other diagnostic modalities. We found a low PPV when we measured the PG I/II ratio in the population with a low prevalence of atrophic gastritis; although the test had a high sensitivity and specificity [52].

In general, we found that the prevalence of atrophic gastritis in Indonesia was low, similar to our previous study [16] and in line with the incidence of gastric cancer. For example, an atrophic score >1 was observed in the antrum in only six patients and in the corpus in only one patient (Table 2), and 74.3% of patients had nonatrophic gastritis (Table 3). The next analysis based on ethnic groups revealed that the PG I/II ratios were significantly lower in subjects from Bangli than in those from Makassar and Surabaya. It is suggested that inhabitants of Bangli have a higher risk of gastric mucosal diseases than the two other ethnic groups. Although these results were in concordance with the prevalence of *H*. *pylori* infection among the ethnic groups, in this study, we analyzed PG levels only in *H*. *pylori-*negative patients. In fact, smoking but not NSAID use and alcohol drinking was significantly higher in subjects from Makassar and Bangli than in those from Surabaya. Dietary habits such as consumption of fermented fish, local spicy sauce, seafood, and fresh fruits may be risk factors, as reported in the Thai population [53].

Similar findings were also observed in the distribution of the four groups according to the ABC method. Subjects from Bangli had the highest proportion of group C and the lowest proportion of group A compared with those from Makassar and Surabaya. However, group D was the lowest in subjects from Bangli compared with the other groups. The ability of the ABC method to discriminate groups at a high risk for the development of gastric cancer was reported in a study on 8286 Japanese patients who underwent endoscopic screening for gastric cancer [4]. The authors suggested that the risk of gastric diseases is very low in group A; the risk of peptic ulcer is elevated in group B; and groups C and D had a higher risk of developing diseases resulting from atrophy of the gastric mucosa, such as gastric cancer, gastric adenoma, and hyperplastic polyps. The risk of gastric cancer is the highest in group D, followed by groups C, B, and A [4]. There are several problems in adopting the ABC method for screening for primary gastric cancer in Indonesia. First, in addition to the problem about the best cutoff for the kits, the criteria were developed for the Japanese population, which has an around 10 times higher incidence of gastric cancer than the Indonesian population (ASR, 29.9 in 100,000). Therefore, Japan has a higher prevalence of atrophic gastritis and the ABC method has a greater accuracy to exclude Japanese patients positive for atrophic gastritis (high PPV). Second, generally, Indonesia has a low prevalence of *H*. *pylori* infection. Therefore, there is a high possibility that a false-positive patient will be included in group D, the group with the highest risk for gastric cancer. There was no a similar concern in our previous study in a low of gastric cancer risk country, Nepal due to the modest prevalence of *H*. *pylori* infection in this country [32]. A validated PGs levels and ABC method could discriminate gastric cancer risk between two group ethnics in Nepal. In addition, we found that the same kits of anti-*H*. *pylori* IgG reached a sufficient sensitivity and specificity among Nepalese population [32]. Further multicenter studies recruiting subjects on a large scale and from various ethnicities are required to confirm the benefits of the ABC criteria to discriminate atrophic gastritis in the Indonesian population.

The number of samples in this study was relatively low, which certainly becomes a study limitation. In addition, we included patients from three cities in three islands. Therefore, our results cannot be generalized to all of Indonesia. Recently, we are continuing our surveys to add to the sample numbers and expanding our investigations to other islands. Next, we included only dyspeptic patients in our study population, and not members of general population or health subjects. The two previous studies also only included dyspeptic patients [22, 23]. In general, gastric mucosal status was severer in the dyspeptic patients than general population or health subjects. Further studies using PGs in general population are substantial as part of a disease prevention strategy in population with low *H*. *pylori* prevalence, even in Indonesia.

## Conclusions

Validation of indirect methods is essential before their application. We confirmed that serum PG levels are a useful biomarker for determining chronic gastritis, but a modest sensitivity for atrophic gastritis in Indonesia. The ABC method should be used with caution in areas with a low prevalence of *H*. *pylori*.

## Supporting information

S1 Table Socio demographic and serology data. Positivity of ELISA was determined with cut-off point ≥5.5 U/ml with the sensitivity, specificity, positive predictive value, and negative predictive value: 86.7%, 96.8%, 65.0%, and 99.1%, respectively.
(PDF)

## Abbreviations

PGs: pepsinogens
OLGA: Operative Link for Gastritis Assessment
ELISA: enzyme-linked immunosorbent assay
AUC: area under curve
ROC: receiver-operating characteristic
NSAID: nonsteroidal anti-inflammatory drug
PPV: positive predictive value
NPV: negative predictive value
OR: odds ratio
CI: confidence interval
