# Supplementary material for: Gastric mucosal status in populations with a low prevalence of Helicobacter pylori in Indonesia
Source: PLoS One. 2017 May 2;12(5):e0176203. doi: 10.1371/journal.pone.0176203 (PMC5413002; doi:10.1371/journal.pone.0176203)
Supplement: S1 Table — Positivity of ELISA was determined with cut-off point ≥5.5 U/ml with the sensitivity, specificity, positive predictive value, and negative predictive value: 86.7%, 96.8%, 65.0%, and 99.1%, respectively. (PDF) [file pone.0176203.s001.pdf]

S1 Table. ***Helicobacter pylori s*****erology and socio-demographic among three populations**

No	Code	Location	Age	Sex	Ethnic	Endoscopic diagnosis	Smoking	Alcohol	Weight	Height	Histology-IHC	Serology
1	KPG36	Bali	42	Male	Balinese	Gastritis	Yes	No	80	175	Negative	Negative
2	KPG37	Bali	55	Female	Balinese	Gastritis	No	No	55	155	Negative	Negative
3	KPG38	Bali	47	Female	Balinese	Gastritis	No	No	67	155	Negative	Negative
4	KPG39	Bali	38	Female	Balinese	Gastritis	No	No	58	160	Negative	Negative
5	KPG40	Bali	37	Male	Balinese	Gastritis	No	No	70	167	Negative	Negative
6	KPG41	Bali	34	Female	Balinese	Gastritis	No	No	60	158	Positive	Positive
7	KPG42	Bali	54	Female	Balinese	Gastric ulcer	No	No	80	178	Positive	Positive
8	KPG43	Bali	43	Male	Balinese	Gastritis	Yes	No	55	160	Positive	Positive
9	KPG44	Bali	23	Female	Balinese	Gastritis	No	No	50	155	Negative	Negative
10	KPG45	Bali	37	Female	Balinese	Gastritis	No	No	50	155	Negative	Negative
11	KPG46	Bali	28	Male	Balinese	Gastritis	No	No	55	165	Negative	Negative
12	KPG47	Bali	60	Female	Balinese	Gastritis	No	No	56	160	Positive	Positive
13	KPG48	Bali	65	Male	Balinese	Gastritis	Yes	No	50	170	Negative	Negative
14	KPG49	Bali	36	Male	balinese	Gastritis	No	No	50	175	Negative	Negative
15	KPG52	Bali	55	Female	Balinese	Gastritis	No	No	50	155	Negative	Negative
16	KPG53	Bali	64	Male	Balinese	Gastritis	Yes	No	60	160	Negative	Negative
17	KPG54	Bali	26	Male	Balinese	Gastritis	No	No	52	164	Negative	Negative
18	KPG55	Bali	35	Female	Balinese	Gastritis	No	No	66	160	Negative	Negative
19	KPG56	Bali	35	Male	Balinese	Gastritis	Yes	No	64	175	Negative	Negative
20	KPG57	Bali	70	Male	Balinese	Gastritis	Yes	No	57	170	Negative	Negative
21	KPG58	Bali	41	Male	Balinese	Gastritis	No	No	80	174	Negative	Negative
22	KPG59	Bali	39	Female	Balinese	Gastritis	No	No	55	150	Negative	Negative
23	KPG60	Bali	50	Female	Balinese	Gastritis	No	No	46	150	Negative	Negative
24	KPG61	Bali	50	Male	Balinese	Gastritis	Yes	No	75	168	Negative	Negative
25	KPG62	Bali	50	Male	Balinese	Gastritis	No	No	68	170	Negative	Negative

26	KPG63	Bali	46	Male	Balinese	Gastritis	Yes	No	85	175	Negative	Negative
27	KPG64	Bali	42	Male	Balinese	Gastritis	No	No	58	160	Positive	Positive
28	KPG65	Bali	35	Male	Balinese	Gastritis	Yes	Yes	64	160	Negative	Negative
29	KPG66	Bali	70	Male	Balinese	Gastritis	No	No	50	160	Negative	Negative
30	KPG67	Bali	55	Female	Balinese	Gastritis	No	No	50	165	Negative	Negative
31	KPG68	Bali	36	Female	Balinese	Gastritis	No	No	48	155	Negative	Negative
32	KPG69	Bali	60	Female	Balinese	Gastritis	No	No	45	150	Negative	Negative
33	KPG70	Bali	33	Male	Balinese	Gastritis	No	No	60	158	Negative	Negative
34	KPG71	Bali	50	Male	Balinese	Gastritis	Yes	No	51	165	Negative	Negative
35	KPG72	Bali	41	Female	Balinese	Gastritis	No	No	65	155	Negative	Negative
36	KPG73	Bali	67	Male	Balinese	Gastric ulcer	Yes	No	63	165	Positive	Positive
37	KPG74	Bali	34	Male	Balinese	Gastritis	No	No	80	165	Negative	Negative
38	KPG75	Bali	59	Female	Balinese	Gastritis	No	No	45	155	Negative	Negative
39	KPG76	Bali	39	Male	Balinese	Gastritis	No	No	73	170	Negative	Negative
40	KPG77	Bali	23	Female	Balinese	Gastritis	No	No	51	160	Negative	Negative
41	KPG78	Bali	43	Female	Balinese	Gastritis	No	No	59	160	Negative	Negative
42	KPG79	Bali	80	Male	Balinese	Gastritis	No	No	62	170	Negative	Negative
43	KPG80	Bali	26	Female	Balinese	Gastritis	No	No	60	170	Negative	Negative
44	KPG81	Bali	33	Male	Balinese	Gastritis	Yes	Yes	76	165	Negative	Negative
45	KPG82	Bali	35	Male	Balinese	Gastritis	No	No	68	172	Negative	Negative
46	KPG83	Bali	37	Female	Balinese	Gastritis	No	No	60	155	Positive	Positive
47	KPG84	Bali	59	Female	Balinese	Gastritis	No	No	51	155	Negative	Negative
48	KPG85	Bali	40	Female	Balinese	Gastritis	No	No	58	160	Negative	Negative
49	KPG86	Bali	40	Female	Balinese	Gastritis	No	No	52	155	Negative	Negative
50	KPG87	Bali	43	Female	Balinese	Gastritis	No	No	59	152	Negative	Negative
51	KPG88	Bali	36	Male	Balinese	Gastritis	No	No	74	162	Negative	Negative
52	KPG89	Bali	60	Female	Balinese	Gastritis	No	No	70	160	Negative	Positive
53	KPG90	Bali	31	Female	Balinese	Gastritis	No	No	50	150	Negative	Negative

54	KPG91	Bali	34	Male	Balinese	Gastritis	No	No	52	167	Negative	Negative
55	KPG92	Bali	53	Male	Balinese	Gastritis	No	No	50	160	Negative	Negative
56	KPG93	Bali	51	Male	Balinese	Gastritis	Yes	No	67	165	Negative	Negative
57	KPG94	Bali	45	Female	Balinese	Gastritis	No	No	49	152	Negative	Negative
58	KPG95	Bali	60	Male	Balinese	Gastritis	No	No	59	160	Negative	Negative
59	KPG96	Bali	51	Female	Balinese	Gastritis	No	No	70	165	Negative	Negative
60	SBY215	Surabaya	37	Male	Javanese	Gastritis	No	No	60	175	Negative	Negative
61	SBY216	Surabaya	31	Male	Javanese	Gastritis	No	No	78	165	Negative	Negative
62	SBY218	Surabaya	63	Male	Javanese	Gastritis	No	No	78	171	Negative	Negative
63	SBY219	Surabaya	56	Female	Tionghoa	Gastritis	No	No	56	158	Negative	Negative
64	SBY220	Surabaya	24	Female	Javanese	Gastritis	No	No	51	161	Negative	Negative
65	SBY221	Surabaya	33	Male	Javanese	Gastritis	No	No	60	163	Negative	Negative
66	SBY223	Surabaya	34	Male	Javanese	Gastritis	No	No	78	165	Negative	Negative
67	SBY224	Surabaya	33	Male	Tionghoa	Gastritis	No	Yes	85	170	Negative	Negative
68	SBY225	Surabaya	54	Male	Tionghoa	Gastric ulcer	No	No	76	175	Negative	Negative
69	SBY226	Surabaya	40	Male	Tionghoa	Gastritis	No	No	75	178	Negative	Negative
70	SBY228	Surabaya	74	Female	Tionghoa	Gastric ulcer	No	No	54	158	Positive	Positive
71	SBY229	Surabaya	38	Female	Madura	Gastritis	No	No	56	167	Negative	Negative
72	SBY230	Surabaya	39	Female	Javanese	Gastritis	No	No	65	166	Negative	Negative
73	SBY232	Surabaya	38	Female	Kupang	Gastritis	No	No	62	160	Negative	Negative
74	SBY233	Surabaya	61	Female	Javanese	Gastritis	No	No	46	152	Negative	Negative
75	SBY234	Surabaya	26	Male	Tionghoa	Gastritis	No	No	63	171	Negative	Negative
76	SBY235	Surabaya	44	Male	Kutai	Gastritis	Yes	Yes	79	162	Negative	Negative
77	SBY236	Surabaya	64	Male	Javanese	Gastritis	No	Yes	67	160	Negative	Negative
78	SBY237	Surabaya	44	Male	Javanese	Gastritis	No	No	67	180	Negative	Negative
79	SBY238	Surabaya	52	Female	Tionghoa	Gastritis	No	No	60	150	Negative	Negative
80	SBY239	Surabaya	54	Male	Javanese	Gastritis	Yes	Yes	70	165	Negative	Negative
81	SBY240	Surabaya	44	Female	Tionghoa	Gastritis	No	No	67	159	Negative	Negative

82	SBY241	Surabaya	69	Male	Tionghoa	Gastritis	No	No	76	178	Negative	Negative
83	SBY243	Surabaya	32	Female	Javanese	Gastritis	No	No	50	163	Negative	Negative
84	SBY244	Surabaya	58	Female	Javanese	Gastritis	No	No	63	156	Negative	Negative
85	SBY245	Surabaya	28	Male	Tionghoa	Gastritis	No	No	60	175	Negative	Negative
86	SBY246	Surabaya	42	Female	Javanese	Gastritis	No	No	40	156	Negative	Negative
87	SBY247	Surabaya	43	Female	Tionghoa	Gastritis	No	No	53	159	Negative	Negative
88	SBY248	Surabaya	59	Male	Tionghoa	Gastritis	No	No	62	160	Negative	Negative
89	SBY249	Surabaya	46	Female	Tionghoa	Gastritis	No	No	70	170	Negative	Negative
90	SBY250	Surabaya	41	Male	Javanese	Gastritis	No	No	67	157	Negative	Negative
91	SBY251	Surabaya	42	Female	Javanese	Gastritis	No	No	55	167	Negative	Negative
92	SBY252	Surabaya	57	Male	Javanese	Gastritis	No	No	70	180	Negative	Negative
93	SBY253	Surabaya	63	Female	Javanese	Gastritis	No	No	56	150	Negative	Negative
94	SBY254	Surabaya	46	Male	Javanese	Gastritis	Yes	No	95	170	Negative	Negative
95	SBY255	Surabaya	48	Male	Tionghoa	Gastritis	No	No	52.4	173	Negative	Negative
96	SBY256	Surabaya	56	Male	Tionghoa	Gastritis	No	No	70	168	Negative	Negative
97	SBY257	Surabaya	37	Male	Tionghoa	Gastritis	Yes	Yes	70	169	Negative	Negative
98	SBY258	Surabaya	47	Male	Javanese	Gastritis	No	No	64	170	Negative	Negative
99	SBY259	Surabaya	70	Male	Javanese	Gastritis	No	No	59	167	Negative	Negative
100	SBY261	Surabaya	51	Male	Javanese	Gastritis	Yes	No	60	160	Negative	Negative
101	SBY262	Surabaya	41	Male	Tionghoa	Gastritis	No	Yes	75	170	Negative	Negative
102	SBY263	Surabaya	70	Female	Tionghoa	Gastritis	No	No	45	147	Negative	Negative
103	SBY264	Surabaya	65	Female	Tionghoa	Gastritis	No	No	56	162	Negative	Negative
104	SBY265	Surabaya	42	Female	Madura	Gastritis	No	No	55	145	Negative	Negative
105	SBY266	Surabaya	14	Female	Javanese	Gastritis	No	No	45	150	Negative	Negative
106	SBY267	Surabaya	39	Male	Javanese	Gastritis	No	No	69	178	Negative	Negative
107	SBY268	Surabaya	30	Male	Tionghoa	Gastritis	No	No	87	170	Negative	Negative
108	SBY270	Surabaya	38	Male	Javanese	Gastritis	Yes	No	86	165	Negative	Negative
109	SBY271	Surabaya	55	Male	kalimantan	Gastritis	No	No	70	163	Negative	Negative

110	SBY272	Surabaya	38	Male	kalimantan	Gastritis	No	No	65	156	Negative	Negative
111	SBY273	Surabaya	31	Female	Javanese	Gastric ulcer	No	No	45	155	Negative	Negative
112	SBY274	Surabaya	34	Female	Javanese	Gastritis	No	No	56	160	Negative	Negative
113	SBY275	Surabaya	62	Male	Tionghoa	Gastritis	No	No	82	180	Negative	Negative
114	SBY276	Surabaya	24	Female	Javanese	Gastritis	No	No	53	165	Negative	Negative
115	SBY277	Surabaya	32	Female	Tionghoa	Gastritis	No	No	44	150	Negative	Negative
116	SBY278	Surabaya	53	Female	Javanese	Gastritis	No	No	60	160	Negative	Negative
117	SBY279	Surabaya	53	Female	kalimantan	Gastritis	No	No	65	160	Negative	Negative
118	SBY280	Surabaya	47	Female	Sulawesi	Gastritis	No	No	71	150	Negative	Negative
119	SBY282	Surabaya	35	Female	Javanese	Gastritis	No	No	64	160	Negative	Negative
120	SBY283	Surabaya	48	Male	Tionghoa	Gastritis	No	No	60	170	Positive	Positive
121	SBY284	Surabaya	30	Male	Javanese	Gastritis	Yes	No	98	177	Negative	Negative
122	SBY285	Surabaya	33	Male	Javanese	Gastritis	No	No	69	172	Negative	Negative
123	SBY286	Surabaya	68	Male	Tionghoa	Gastritis	No	No	63	165	Negative	Negative
124	SBY287	Surabaya	55	Male	Javanese	Gastritis	No	No	71	170	Negative	Negative
125	SBY288	Surabaya	49	Female	Javanese	Gastritis	No	No	73	151	Negative	Negative
126	SBY289	Surabaya	55	Male	Arab	Gastritis	Yes	No	95	180	Positive	Negative
127	SBY290	Surabaya	38	Female	Javanese	Gastritis	No	No	55	156	Negative	Negative
128	SBY291	Surabaya	32	Female	Madura	Gastritis	No	No	55	158	Negative	Negative
129	SBY292	Surabaya	61	Female	Javanese	Gastritis	No	No	70	150	Negative	Negative
130	SBY293	Surabaya	61	Female	Kalimantan	Gastritis	No	No	57	154	Negative	Negative
131	SBY294	Surabaya	54	Male	Javanese	Gastric ulcer	No	No	67	165	Negative	Negative
132	SBY295	Surabaya	50	Female	Sulawesi	Gastritis	Yes	No	63	156	Negative	Negative
133	SBY296	Surabaya	37	Male	Javanese	Gastritis	No	No	65	172	Negative	Negative
134	SBY297	Surabaya	46	Female	Tionghoa	Gastritis	No	No	51	158	Negative	Negative
135	SBY298	Surabaya	34	Female	Javanese	Gastritis	No	No	40	155	Negative	Negative
136	SBY299	Surabaya	35	Female	Javanese	Gastritis	No	No	59	155	Negative	Negative
137	SBY300	Surabaya	39	Female	Javanese	Gastritis	No	No	37	155	Negative	Negative

138	SBY302	Surabaya	39	Female	Javanese	Gastritis	No	No	77	160	Negative	Negative
139	SBY303	Surabaya	43	Female	Javanese	Gastritis	No	No	75	160	Negative	Negative
140	SBY304	Surabaya	56	Female	Ambonese	Gastritis	No	No	63	168	Positive	Positive
141	SBY305	Surabaya	46	Female	N/A	Gastritis	No	No	N/A	N/A	Negative	Negative
142	SBY306	Surabaya	51	Female	Javanese	Gastritis	No	No	78	160	Negative	Negative
143	SBY307	Surabaya	53	Male	Javanese	Gastritis	No	No	54	155	Negative	Negative
144	SBY308	Surabaya	52	Female	Javanese	Gastritis	No	No	54	150	Negative	Negative
145	SBY309	Surabaya	23	Male	kalimantan	Gastritis	No	No	45	165	Negative	Negative
146	SBY310	Surabaya	51	Male	Tionghoa	Gastritis	No	No	56	173	Negative	Negative
147	SBY311	Surabaya	55	Male	Javanese	Gastritis	No	No	46	160	Negative	Negative
148	SBY313	Surabaya	51	Female	Javanese	Gastritis	No	No	40	150	Negative	Negative
149	SBY315	Surabaya	57	Female	Javanese	Gastritis	No	No	54	153	Negative	Negative
150	SBY316	Surabaya	35	Female	Javanese	Gastritis	No	No	51	153	Negative	Negative
151	SBY317	Surabaya	64	Male	Javanese	Gastritis	No	No	54	167	Negative	Negative
152	SBY318	Surabaya	58	Female	Javanese	Gastritis	No	No	58	154	Negative	Negative
153	SBY319	Surabaya	44	Female	Sulawesi	Gastritis	No	No	45	150	Negative	Negative
154	SBY320	Surabaya	44	Male	Irian	Gastritis	No	No	57	165	Negative	Negative
155	SBY321	Surabaya	57	Male	Javanese	Peptic ulcer	No	No	61	160	Negative	Negative
156	SBY322	Surabaya	53	Male	Javanese	Gastritis	No	No	70	155	Negative	Negative
157	SBY323	Surabaya	50	Male	Javanese	Gastritis	No	No	78	158	Negative	Negative
158	SBY324	Surabaya	58	Male	Javanese	Gastritis	Yes	No	65	178	Negative	Negative
159	001 G	Makassar	49	Male	Toraja	Gastritis	No	No	70	165	Negative	Negative
160	003 G	Makassar	45	Female	Toraja	Gastritis	No	No	53	150	Negative	Negative
161	005 G	Makassar	54	Male	Mandar	Gastritis	No	No	64	160	Negative	Negative
162	006 G	Makassar	40	Male	Ternate	Gastritis	No	Yes	60	164	Negative	Negative
163	007 G	Makassar	39	Male	Tolaki	Gastritis	No	No	60	170	Negative	Negative
164	009 G	Makassar	18	Female	Makassar	Gastritis	No	No	41	156	Negative	Negative
165	010 G	Makassar	39	Female	Bugis	Gastritis	No	No	36	150	Negative	Negative

166	011 G	Makassar	52	Female	Toraja	Peptic ulcer	No	No	78	156	Negative	Negative
167	012 G	Makassar	82	Female	Ambonese	Gastritis	No	No	39	150	Negative	Negative
168	013 G	Makassar	57	Female	Makassar	Gastritis	No	No	49	150	Negative	Negative
169	016 G	Makassar	46	Male	Makassar	Gastritis	No	No	65	173	Negative	Negative
170	017 G	Makassar	46	Male	Bugis	Gastritis	No	No	52	165	Negative	Negative
171	018 G	Makassar	49	Female	Bugis	Gastritis	No	No	50	152	Negative	Negative
172	020 G	Makassar	45	Female	Toraja	Gastritis	No	No	57	152	Positive	Positive
173	022 G	Makassar	57	Male	Bugis	Gastritis	Yes	Yes	50	173	Negative	Negative
174	023 G	Makassar	53	Male	Bugis	Gastritis	Yes	No	50	165	Negative	Negative
175	025 G	Makassar	41	Male	Ternate	Gastritis	No	No	60	165	Negative	Negative
176	026 G	Makassar	51	Male	Tolaki	Gastritis	Yes	No	60	154	Negative	Negative
177	027 G	Makassar	56	Female	Flores	Gastritis	No	No	50	153	Negative	Negative
178	028 G	Makassar	55	Female	Papua	Gastritis	No	No	N/A	N/A	Negative	Negative
179	029 G	Makassar	41	Male	Bugis	Gastritis	No	No	58	180	Positive	Positive
180	030 G	Makassar	50	Male	Mandar	Gastritis	No	No	70	160	Negative	Negative
181	032 G	Makassar	54	Female	Bugis	Gastritis	No	No	48	150	Negative	Negative
182	033 G	Makassar	69	Male	Bugis	Gastritis	No	No	60	154	Negative	Negative
183	034 G	Makassar	53	Female	Palopo	Gastritis	No	No	56	154	Negative	Negative
184	035 G	Makassar	57	Female	Kaili	Gastritis	No	No	58	154	Negative	Negative
185	036 G	Makassar	54	Female	Makassar	Gastritis	No	No	60	150	Negative	Negative
186	037 G	Makassar	17	Female	Mandar	Gastritis	No	No	36	153	Negative	Negative
187	039 G	Makassar	63	Male	Saluan	Gastritis	No	No	53	164	Negative	Negative
188	041 G	Makassar	26	Male	Mandar	Gastritis	No	No	48	155	Negative	Negative
189	042 G	Makassar	38	Male	Makassar	Gastritis	Yes	No	50	160	Negative	Negative
190	043 G	Makassar	42	Female	Bugis	Gastritis	No	No	40	155	Negative	Negative
191	044 G	Makassar	51	Male	Banjar	Gastritis	Yes	No	68	170	Negative	Negative
192	045 G	Makassar	45	Male	Bugis	Gastritis	Yes	No	60	170	Negative	Negative

193	046 G	Makassar	33	Male	Bugis	Gastritis	No	No	60	165	Negative	Negative
194	048 G	Makassar	46	Female	Bugis	Gastritis	No	No	45	150	Negative	Negative
195	049 G	Makassar	23	Male	Bugis	Gastritis	No	No	51	160	Negative	Negative
196	050 G	Makassar	54	Female	Tolaki	Gastritis	No	No	45	155	Negative	Positive
197	051 G	Makassar	37	Female	Bugis	Gastritis	No	No	50	153	Negative	Negative
198	052 G	Makassar	27	Male	Luwu	Gastritis	Yes	No	77	167	Negative	Negative
199	055 G	Makassar	24	Male	Makassar	Gastritis	No	No	54	165	Negative	Negative
200	057 G	Makassar	40	Male	Bugis	Gastritis	Yes	Yes	49	165	Negative	Negative
201	059 G	Makassar	48	Female	Mandar	Gastritis	No	No	39	140	Negative	Negative
202	060 G	Makassar	53	Male	Bugis	Gastritis	Yes	No	55	160	Negative	Negative
203	061 G	Makassar	40	Female	N/A	Gastritis	No	No	41	151	Negative	Negative
204	062 G	Makassar	63	Male	Bugis	Gastritis	No	No	68	170	Negative	Negative
205	063 G	Makassar	38	Male	Bugis	Gastritis	No	No	60	160	Negative	Negative
206	064 G	Makassar	41	Female	Makassar	Gastritis	No	No	47	150	Negative	Negative
207	066 G	Makassar	42	Female	Kendari	Gastritis	No	No	55	150	Negative	Negative
208	067 G	Makassar	52	Female	Bugis	Gastritis	No	No	50	150	Negative	Negative
209	068 G	Makassar	50	Male	Bugis	Gastritis	Yes	Yes	60	170	Negative	Negative
210	069 G	Makassar	24	Male	Bugis	Gastritis	Yes	Yes	50	165	Negative	Positive
211	070 G	Makassar	43	Female	Tolaki	Gastritis	No	No	65	155	Negative	Negative
212	071 G	Makassar	50	Male	Poso	Gastritis	Yes	Yes	72	168	Negative	Negative
213	073 G	Makassar	62	Male	Toraja	Gastritis	Yes	No	44	155	Negative	Positive
214	074 G	Makassar	45	Female	Bugis	Gastritis	No	No	44	156	Negative	Negative
215	075 G	Makassar	39	Female	Toraja	Gastritis	No	No	54	168	Negative	Positive
216	076 G	Makassar	69	Male	Bugis	Gastritis	No	No	45	152	Negative	Negative
217	077 G	Makassar	32	Male	Tolaki	Gastritis	Yes	Yes	67	163	Negative	Negative
218	078 G	Makassar	45	Male	Tolaki	Gastritis	Yes	No	71	168	Negative	Negative
219	079 G	Makassar	43	Male	Bugis	Gastritis	Yes	No	70	165	Positive	Negative

220	080 G	Makassar	52	Male	Bugis	Gastritis	No	No	70	162	Negative	Negative
221	081 G	Makassar	37	Male	Bugis	Gastritis	No	No	62	165	Negative	Negative
222	001 PU	Makassar	45	Male	Toraja	Gastritis	No	No	54	160	Positive	Positive
223	002 PU	Makassar	47	Female	Papua	Gastric ulcer	No	No	72	150	Negative	Negative
224	003 PU	Makassar	34	Male	Toraja	Gastric ulcer	Yes	Yes	73	165	Negative	Negative
225	004 PU	Makassar	55	Male	Toraja	Gastric ulcer	Yes	Yes	53	165	Negative	Positive
226	005 PU	Makassar	54	Female	Luwu	Gastric ulcer	No	No	44	150	Negative	Negative
227	006 PU	Makassar	48	Female	Buton	Gastric ulcer	No	No	65	154	Negative	Negative
228	008 PU	Makassar	55	Male	Makassar	Gastric ulcer	No	No	65	167	Negative	Positive
229	009 PU	Makassar	52	Female	Tidore	Gastric ulcer	No	No	59	155	Negative	Negative
230	010 PU	Makassar	37	Female	Bugis	Gastric ulcer	No	No	45	150	Negative	Negative
231	011PU	Makassar	54	Male	Makassar	Gastric ulcer	No	No	50	150	Negative	Negative
232	012 PU	Makassar	67	Male	Bugis	Gastric ulcer	Yes	No	45	160	Negative	Negative
233	013 PU	Makassar	69	Male	Bugis	Gastric ulcer	Yes	No	55	165	Negative	Negative
